# Current Status of Novel Multifunctional Targeted Pt(IV) Compounds and Their Reductive Release Properties

**DOI:** 10.3390/molecules29040746

**Published:** 2024-02-06

**Authors:** Lingwen Xu, Xiangyu Kong, Xinzhi Li, Bin Zhang, Yuxiao Deng, Jinhu Wang, Chonggang Duan, Daizhou Zhang, Wentao Liu

**Affiliations:** 1 Institute of Chemical Drugs, Shandong Academy of Pharmaceutical Sciences, Jinan 250101, China; lingwenxu2022@163.com (L.X.); kongxiangyu@sdaps.cn (X.K.); lixinzhi@sdaps.cn (X.L.); zhangbin@sdaps.cn (B.Z.); dengyuxiao1124@163.com (Y.D.); wjh1573@126.com (J.W.); dcg505@163.com (C.D.); 2 Shandong Provincial Key Laboratory of Biopharmaceuticals, Shandong Academy of Pharmaceutical Sciences, Jinan 250101, China

**Keywords:** divalent platinum, tetravalent platinum, multifunctional targeted, anti-tumor, reductive release

## Abstract

Platinum-based drugs are widely used in chemotherapy for various types of cancer and are considered crucial. Tetravalent platinum (Pt(IV)) compounds have gained significant attention and have been extensively researched among these drugs. Traditionally, Pt(IV) compounds are reduced to divalent platinum (Pt(II)) after entering cells, causing DNA lesions and exhibiting their anti-tumor effect. However, the available evidence indicates that some Pt(IV) derivatives may differ from the traditional mechanism and exert their anti-tumor effect through their overall structure. This review primarily focuses on the existing literature regarding targeted Pt(II) and Pt(IV) compounds, with a specific emphasis on their in vivo mode of action and the properties of reduction release in multifunctional Pt(IV) compounds. This review provides a comprehensive summary of the design and synthesis strategies employed for Pt(II) derivatives that selectively target various enzymes (glucose receptor, folate, telomerase, etc.) or substances (mitochondria, oleic acid, etc.). Furthermore, it thoroughly examines and summarizes the rational design, anti-tumor mechanism of action, and reductive release capacity of novel multifunctional Pt(IV) compounds, such as those targeting p53-MDM2, COX-2, lipid metabolism, dual drugs, and drug delivery systems. Finally, this review aims to provide theoretical support for the rational design and development of new targeted Pt(IV) compounds.

## 1. Introduction

**Figure 1:**
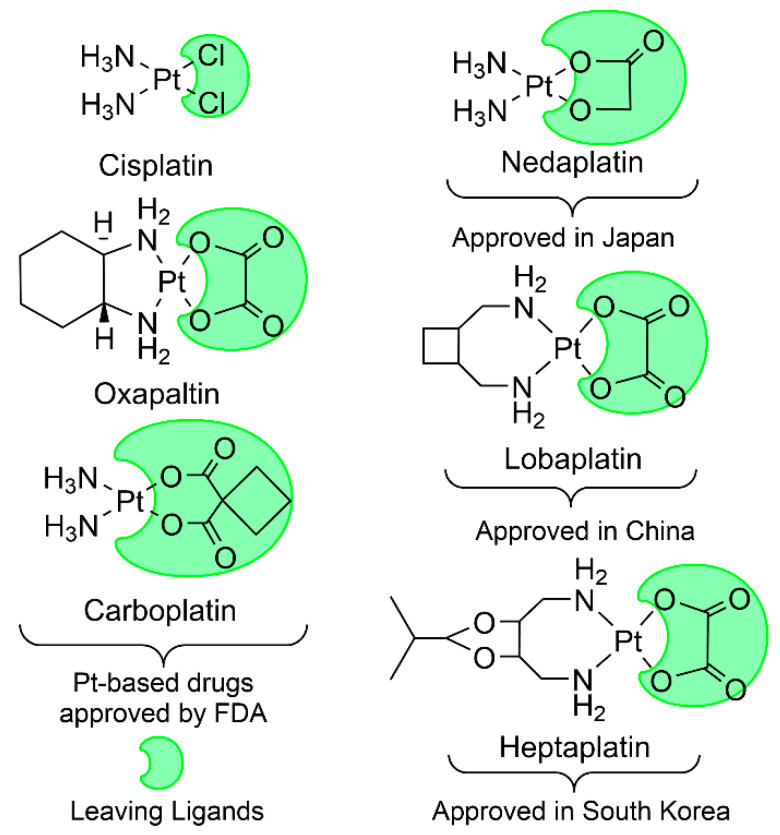
Chemical structures of classical platinum-based drugs.

**Figure 2:**
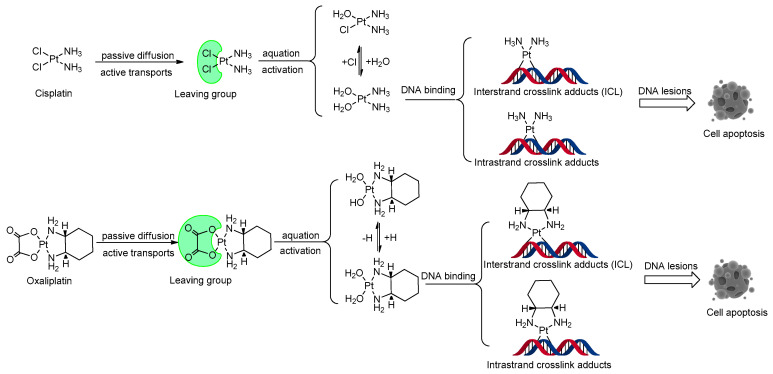
Flowchart of the mechanism of anti-tumor action of cisplatin and oxaliplatin as an example. The green shadow is the leaving groups.

With the advancement of medical technology, there is a diversified development trend in the treatment of tumors. This includes developing and innovating surgical treatment, radiotherapy, immunotherapy, and chemotherapy. Classical platinum-based drugs, such as cisplatin, oxaliplatin, and carboplatin, are crucial anti-cancer agents (Figure 1) [1,2,3]. These drugs primarily act against tumors in three ways: (1) DNA cross-linking: Pt(II) drugs enter cells through passive diffusion, assisted by active transport mechanisms involving copper transporter (Ctr1) and organic cation transporter (OCT2), among others [3,4]. Once inside tumor cells, platinum drugs form covalent DNA cross-links with nuclear DNA, disrupting its structure and function. Consequently, this impedes DNA repair and replication processes. The hydrolytic activation of Pt(II) drugs leads to an increase in the formal charge on the Pt(II) center. This increase enhances the reactivity of the Pt(II) center towards nucleophilic sites in DNA. Among these nucleophilic sites, the N7 atoms of the purine residues guanine and adenine are particularly susceptible to platinization. The preferential platinization of these N7 atoms leads to DNA strand breaks, which in turn disrupt the normal growth and division of tumor cells (Figure 2) [4,5,6,7,8]. (2) Gene transcriptional and transcription factor inhibition: Pt(II) can disrupt gene transcription and transcription factor activity. Certain Pt(II) compounds can also bind to DNA and prevent the normal functioning of RNA polymerase, impeding the synthesis of RNA and the transcription process of genes [3,9]. (3) Oxidative stress and free radical generation: Oxidative stress is a significant mechanism through which Pt(II) compounds exert their anti-tumor effects. These compounds bind to and consume intracellular reducing substances and form complexes with mitochondrial DNA, causing mitochondrial dysfunction and an increase in the production of reactive oxygen species (ROS). This, in turn, induces oxidative stress and ultimately leads to tumor cell death. Additionally, ROS can upregulate the expression of transporters for Pt(II) compounds, such as Ctr1, enhance cellular DNA damage repair, increase the protein level and transcriptional activity of nuclear factor (erythroid-derived 2)-like 2 (Nrf2) and hypoxia-inducible factor-1α (HIF-1α), and regulate the dynamic activity of mitochondria [10,11].

However, the application and development of platinum-based drugs are currently limited due to tumor resistance and severe toxic side effects. These side effects include neurotoxicity, nephrotoxicity, ototoxicity, and gastrointestinal toxicity. It is worth noting that approximately 80% of patients with epithelial ovarian cancer experience tumor recurrence and chemotherapy failure due to resistance to platinum-based drugs [12,13,14]. Additionally, Pt(II) regimens require intravenous injection for drug administration, as oral administration is ineffective and inconvenient for patients.

Pt(IV), the oxidized state of divalent platinum, is a focal point in the research and development of novel platinum-based drugs. Pt(IV) compounds offer several advantages: (1) Improved drug stability: Pt(IV) compounds exhibit good stability, reducing drug degradation and inactivation in the body, thus prolonging the drug’s half-life [13,15,16,17]. (2) Increased selectivity: Pt(IV) compounds can selectively target specific tumors by tailoring their ligand structure and chemical properties, minimizing toxicity to normal cells [18]. (3) Targeted delivery: Pt(IV) compounds can achieve targeted drug delivery through ligands or nanocarriers, enhancing their accumulation in tumor cells [18,19].

The utilization of tetravalent oxoplatin has led to the development of novel Pt(IV)-small molecule conjugates, which have addressed the limitations of traditional platinum drugs and show potential in reducing toxic side effects. Classical Pt(II) compounds are susceptible to non-selective ligand substitution. For instance, approximately 90% of cisplatin irreversibly binds to plasma proteins, leading to the drug’s inactivation in the bloodstream. Additionally, only 1% of cisplatin binds to nuclear DNA, which contributes to the development of tumor resistance [20,21,22]. However, Pt(IV) compounds with their pseudooctahedral geometry exhibit greater inertness to ligand substitution compared to Pt(II) complexes, reducing isolation and deactivation of platinum drugs during transport to tumor cells and enhancing blood stability [23]. By utilizing hydroxyl groups in axial positions, Pt(IV) compounds allow for fine-tuning desired biological properties, such as lipophilicity, redox stability, targeting of cancer cells, orthogonal or complementary biological activity, and improved cellular uptake. In contrast to the four-coordinate Pt(II) compounds, the low-spin d^6^ octahedral complex structure tends to be saturated, displaying higher kinetic inertness to ligand substitution reactions. This minimizes adverse reactions with biomolecules (e.g., undesired interactions between Pt(II) and proteins or intracellular thiols) before DNA binding occurs [3,24,25,26,27].

Classic platinum drugs, such as cisplatin, exert their mechanism of action by entering the human body and transforming into hydroxy complexes and hydrates [28,29,30]. These platinum hydroxy complexes or hydrates then form chelation rings by binding to adjacent guanine or adenine bases in the DNA single strand (1,2-GPG or 1,3-APG) [4,29,31]. The formation of these chelation rings disrupts the hydrogen bonds between DNA double strands, thereby disturbing the DNA double helix structure in tumor cells. This disruption hinders the normal replication of tumor cells and induces apoptosis [32,33]. The binding of cisplatin to DNA primarily occurs through intrastrand cross-links, with approximately 90–95% of 1,2-GPG and 1,3-APG intrastrand cross-links and 5–8% interstrand cross-link (ICL) products [4,33,34]. Traditionally, Pt(IV) pro-drugs, with their inert octahedral configuration, require activation through intracellular reducing agents such as glutathione (GSH), ascorbic acid (ASA), and other reductants in order to transform into Pt(II) monofunctional and bifunctional hydrate forms. Only then can they interact with DNA, form DNA adducts, and exert their anti-cancer activity, generating biological effects similar to classical platinum drugs [35,36]. However, there is evidence suggesting that the mechanism of action between Pt(II) and Pt(IV) compounds (especially double axial reaction products) on DNA is not identical after entering cells [37,38,39,40]. Therefore, further investigation is necessary to better understand the anti-tumor action of novel Pt(IV) compounds derived from small molecules and platinum compounds, including their specific form of action and underlying mechanism. This review article provides a comprehensive summary of the design and synthesis of classical platinum compounds and their targeted derivatives, along with the evaluation of their anti-tumor bioactivity. Additionally, it examines the in vivo and in vitro reductive action to explore platinum compounds specific mechanisms of action further.

## 2. Novel Multifunctional Targeted Pt(II) Compounds

In recent years, significant progress has been in modifying Pt(II) compounds, leading to the synthesis and study of thousands of compounds for their pharmacological activities. Extensive research has been conducted on platinum derivatives that selectively target specific enzymes and functional proteins. The design and synthesis of platinum compounds with specific targets have become a prominent area of research. In this section, we aim to summarize the research efforts focused on Pt(II) compounds that target specific enzymes or substances.

### 2.1. Targeting Glucose Receptors with Pt(II) Compounds

**Figure 3:**
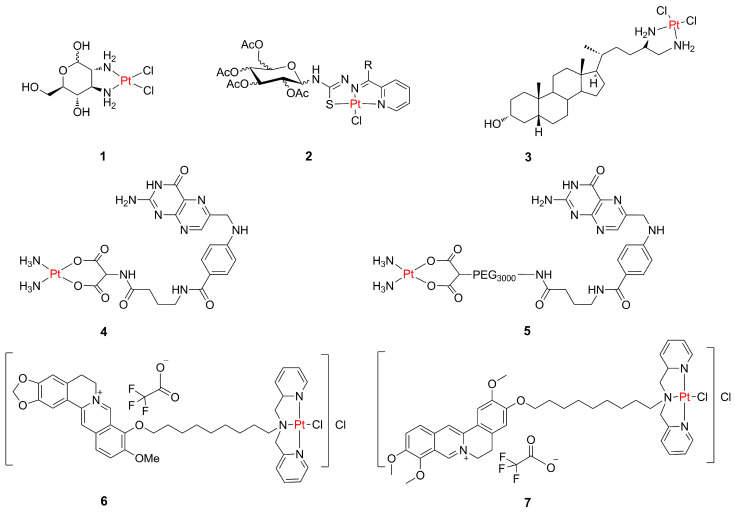
Chemical structures of novel targeting Pt(II) compounds. Red font represents core platinum atoms.

Malignant tumor cells have a high glucose uptake as an energy source for cell division. Similar to the Warburg effect, changes in the metabolic state of tumor cells result in an excessive demand for glucose. This increased glucose uptake is associated with the over-expression of glucose membrane transporters (e.g., GLUT1-4) in the tumor cells [41]. Recent studies have synthesized new Pt(II) compounds with glucose-targeting properties by replacing amino ligands with amino sugars. These Pt(II) derivatives were created using 2,3-diaminosugars as the starting materials. These compounds have shown promising activity in vitro and animal models by inhibiting the uptake of glucose by tumor cells. Compound **1** (PtCl_2_ (2,3-diamino-2,3-dideoxy-D-glucose)) exhibited significant activity in vitro and animal models, not only inhibiting glucose uptake by tumor cells but also prolonging the survival time of nude mice at a dose of 50 mg/kg^−1^ intraperitoneally (Figure 3). Although cisplatin showed similar effects at a lower dose (8 mg/kg^−1^), this dose was close to the maximum tolerated dose (MTD) of 13 mg/kg^−1^. Research on various glucose transporter inhibitors has confirmed that cell uptake relies on glycosylation, which directly affects cell cytotoxicity [42].

In a separate study, a group of researchers synthesized eight novel dual-targeting Pt(II) compounds that specifically targeted GLUT1 and Pgp proteins. They extensively explored the in vitro anti-cancer mechanisms of these glycosylated thiosemicarbazone Pt(II) complexes, which acted as substrates for both GLUT1 and Pgp, potentially contributing to targeted therapy. Cytotoxicity experiments showed that compound **2** displayed a broad spectrum of anti-tumor activity against four different cancer cell lines, with significant cytotoxic effects on A549 cells (Compound **2**, IC_50_ value (∼10 μM); cisplatin, IC_50_ value (∼17 μM)). Inhibition experiments using genistein and EDG confirmed the GLUT1-dependent nature of compound **2** (Figure 3). Cellular uptake experiments demonstrated that the transport characteristics of GLUT1 improved the uptake of Pt(II) into tumor cells. Compound **2** accumulated rapidly in cells, with an accumulation rate of 23.15 ng, which was 2.3-fold higher than that of cisplatin (10.105 ng) at 6 h. Through GLUTs, compound **2** quickly localized to lysosomes upon cell entry, increased intracellular ROS levels, and induced apoptosis through lysosomal damage. The cellular thermal shift assay (CETSA) indicated that GLUT1 and Pgp proteins were potential key targets of compound **2**, suggesting the potential for developing both GLUT1 and Pgp as transport proteins. Furthermore, the stability of the dual-targeting Pt(II) complex in human blood was enhanced by binding to BSA during the drug delivery process. Therefore, these glycosylated Pt(II) complexes exhibited dual targeting properties, enhancing their tumor-targeting efficacy [43].

### 2.2. Targeting of Bile Acids with Pt(II) Compounds

Complexes bound by Pt(II) ligands can transport bile acids, known as cholesteryl esters, to the liver. Transporter proteins facilitate this transportation on the surface of liver cells that take up bile salt ions from the serum [44,45]. A novel oral cancer drug has been developed, where the active component is a bile acid molecule paired with cis-diaminoplatinum(II), functioning as a chelator [46]. Initial in vitro assays have shown its effectiveness in cultured mouse liver cancer cells. Additionally, the complex has been confirmed to have anti-tumor activity in a homozygous in situ rat model of hepatocellular carcinoma. Another approach to linking bile acids to the Pt(II) center involves conjugation with a non-dissociative group ligand, resulting in complexes that exhibit activity in cultured cells and have a mechanism of action similar to cisplatin [47]. A different study synthesized a series of cholic acid-derived 1,2- and 1,3-diamines and their Pt(II) complexes to combine two bioactive moieties and create compounds with highly cytotoxic properties [48]. The synthesized compounds were evaluated for in vitro bioactivity, and some of them showed significant anti-tumor activity. Compound **3** demonstrated only moderate cytotoxicity (IC_50_: 28 μM) in an in vitro activity test against the cervical cancer cell line HeLa, but it showed no toxicity towards normal fibroblasts (Figure 3). This selectivity offers possibilities for modifying the complex to enhance its efficacy while maintaining non-toxicity toward healthy cells.

### 2.3. Targeting Folate Receptor with Pt(II) Compounds

Folate receptor (FR) glycoprotein is known to be overexpressed in various tumor cell lines [49,50]. Folate, which contains a pterin unit, plays a crucial role in several central biochemical pathways, including those related to DNA synthesis [51]. Due to the increased folate uptake by cancer cells in response to rapid growth demands, the folate-binding motif has become a target for platinum complexes. While sugars and steroids have received more attention, folate has been relatively understudied as a target for Pt(II) complexes [52,53,54]. For instance, early studies on the interaction between cisplatin and cytosolic folate involved isolating the complex formed by replacing the chlorinated ligand with tetrahydrofolate. Although this complex inhibits dihydrofolate reductase and the folate transport system, its lack of unstable coordination sites suggests it cannot act as a cytotoxic agent like cisplatin. In separate studies, researchers have synthesized various cisplatin and carboplatin derivatives with folate moieties conjugated to either the non-dissociative ligand or the dissociative ligand (Figure 3, compound **4**) [55]. Unfortunately, the low water solubility of these compounds has hindered their application in evaluating anti-tumor bioactivity. To address this limitation, PEG was used as a linker between the dicarboxylic acid chelator and the folate moiety (Figure 3, compound **5**) to enhance the water solubility of the compound. Mechanistic studies have shown that this conjugate is taken up through folate receptor-mediated endocytosis. However, the compound exhibits lower levels of DNA binding and forms adducts, making it less active than carboplatin in terms of anti-tumor activity. Furthermore, folate targeting has been successfully utilized to direct platinum-loaded nanoparticle drug delivery vehicles into FR-expressing cancer cells.

### 2.4. Targeting Mitochondria with Pt(II) Compounds

**Scheme 1:**
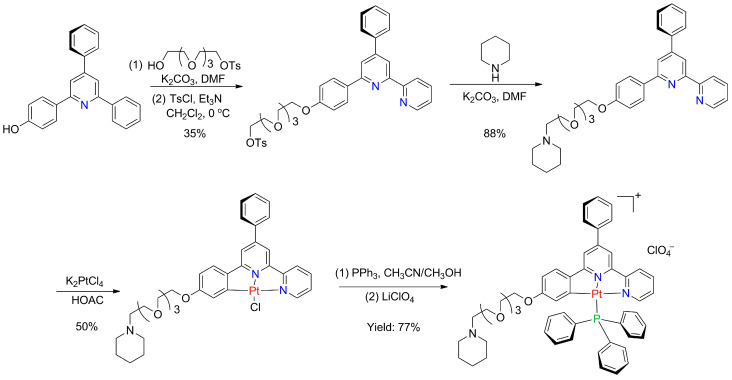
Synthesis route of **PIP-platin**. (Reprinted with permission from Ref. [57]. Copyright 2017 The Royal Society of Chemistry). Nitrogen atoms are shown in blue, platinum atoms are shown in red, and phosphorus atom is shown in green.

Mitochondrial-targeted therapy offers an alternative approach to cancer treatment, specifically addressing the challenges of metastasis and drug resistance commonly encountered in conventional therapies [56]. The cationic cycloplatin Pt(II) complex **PIP-Platin** has been designed and synthesized by researchers to achieve targeted delivery to mitochondria in cancer cells (Scheme 1) [57]. The synthesis process involved several steps. Firstly, a crucial precursor, 4-phenyl-6-phenol-2,2′-bipyridine, was synthesized using an improved Krohnke-type reaction. This precursor efficiently reacted with oligo(ethylene glycol chains), followed by a substitution reaction with piperidine to obtain the corresponding ligand. The ligand then underwent a metal-catalyzed reaction with K_2_PtCl_4_ to form the cyclometalated platinum complex. Finally, the complex underwent ligand exchange with PPh_3_ and salt decomposition with LiClO_4_, resulting in the cationic cycloplatin complex **PIP-Platin**. **PIP-platin** exhibited selective transport and accumulation in mitochondria, exerting toxic effects on various cancer cells. Furthermore, **PIP-platin** has demonstrated effective inhibition of the Wnt signaling pathway by preventing the nuclear translocation of β-catenin, thereby inhibiting cell proliferation. With its significant potential for mitochondrial delivery, **PIP-platin** effectively inhibits tumor cell proliferation and migration/invasion through β-catenin and holds promise as a bifunctional targeted anti-tumor candidate.

### 2.5. Targeting Telomerase with Pt(II) Compounds

Two novel Pt(II) complexes, [Pt(B-TFA)Cl]Cl (**6**) and [Pt(J-TFA)Cl]Cl (**7**), were synthesized for the first time in a study targeting telomerase [58] (Figure 3). These platinum analogs have potential applications in cellular studies as luminescent agents and as potent telomerase inhibitors. They were found to induce apoptosis in bladder T-24 tumor cells by targeting telomerase. The inhibition rates of telomerase in T-24 cells by compounds **6** and **7** were 61.67% and 45%, respectively, compared to B-TFA (4.5%) and J-TFA (31.83%). Additionally, these compounds were observed to induce mitochondrial membrane potential loss, mitochondrial dysfunction, telomeric DNA damage, and cell cycle arrest in tumor cells. This was achieved by upregulating the expression of proteins such as caspase 3, caspase 9, and Apaf-1, and downregulating CDK2, cyclin D1, and BCL-2.

**Scheme 2:**
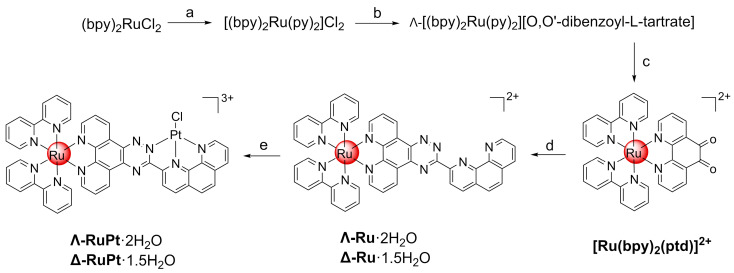
The synthetic routes of Λ-RuPt and Δ-RuPt. Reagents: (a) pyridine, MeOH, H_2_O, reflux; (b) disodium (-)-O,O’-dibenzoyl-LL-tartrate; (c) (i) 1,10-phenanthroline-5,6-dione, ethylene glycol–H_2_O (9:1), 120 °C, (ii) NaClO_4_; H_2_O; (d) 1,10-phenanthroline-2-carboxamide hydrazone, CH_3_CN/EtOH (3:1), alumina/CH_3_CN. (Reprinted with permission from Ref. [59]. Copyright 2002 Elsevier). (e) Pt(DMSO)_2_Cl_2_, MeCN/EtOH, reflux. (Reprinted with permission from Ref. [60]. Copyright 2022 Wiley). Red shading represents ruthenium atoms.

In another study, the synthesis and anti-tumor activity evaluation of two binuclear coordination compounds (**Δ-RuPt** and **Λ-RuPt**) were reported as agents inducing telomerase dysfunction alongside chemotherapy (Scheme 2) [59,60]. These platinum complexes exhibited a high binding affinity for G-quadruplex DNA. They interacted with hTel G4 DNA through π-π stacking and interaction with negatively charged channels and cation-π binding. To enhance subcellular localization within the cell nucleus and provide tumor-targeting properties, biotin-functionalized DNA cages were encapsulated in the metal complexes, forming **Δ-RuPt**@biotin-DNA cage and **Λ-RuPt**@biotin-DNA cage. These nanoparticles showed high selectivity for cancer cells and induced telomeric dysfunction in cisplatin-resistant cancer cells. They also significantly suppressed the growth of cisplatin-resistant lung cancer tumors in A549R tumor-bearing mice. Furthermore, researchers determined the telomere length of A549R cells using single-color multiplex quantitative polymerase chain reaction (qPCR). Treatment with **Δ-RuPt**@Biotin-DNA cage or **Λ-RuPt**@Biotin-DNA cage (1 μM) significantly shortened the telomere length of A549R cells (**Δ-RuPt**@Biotin-DNA Cage: 86.5 ± 2.5%). In Western blot analysis, the downregulation of hTERT and TRF2 expression and the significant upregulation of TRF1 and POT1 expression were consistent with previous reports on telomere dysfunction caused by G-quadruplex DNA binding.

The article focuses on the research direction of targeting units to classical Pt(II) drugs rather than providing a comprehensive overview of all derivatives of Pt(II) compounds. Targeting can also be performed at the subcellular level, directing platinum to specific organelles to trigger different biological effects. These drugs are suitable for platinum-based therapies due to their target-directed structural component and their ability to interact with cancer cells. Additionally, apart from targeting tumor cells themselves, inhibiting tumor cell growth can be achieved by discovering proteins that enable angiogenesis or can be specifically selected in acidic and hypoxic environments

## 3. Novel Multifunctional Targeted Pt(IV) Compounds and Reduction Releasing

**Figure 4:**
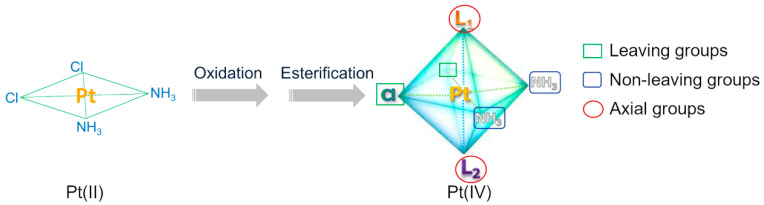
Taking cisplatin as an example, there are changes in the spatial structure of Pt(II) and Pt(IV) compounds.

**Figure 5:**
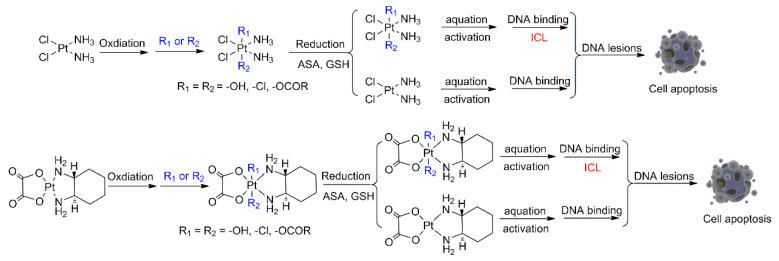
Flowchart of the mechanism of anti-tumor action of Pt(IV) compounds. The blue R1 or R2 font is the substituent group and the red font represents the interstrand cross-linking (ICL).

In recent years, there has been a growing interest in Pt(IV) compounds as a potential solution to side effects, drug resistance, and the administration form of bivalent platinum drugs. Significant progress has been made in the synthesis of novel Pt(IV) pro-drugs that exhibit good activity, high efficiency, and low toxicity. For instance, satraplatin, the first tetravalent platinum compound to undergo clinical trials, was successfully synthesized by Johnson-Mathey in the UK [61,62]. Satraplatin offers a convenient oral route of administration, similar to cisplatin, and functions by cross-linking DNA strands, distorting DNA, and inhibiting DNA transcription and replication. Its ability to overcome cisplatin resistance is attributed to the asymmetric nature of DNA damage, which evades recognition by DNA repair proteins [63]. Furthermore, iproplatin and ormaplatin, both demonstrating superior clinical oncological activity, serve as promising indicators for the development of novel tetravalent platinum-based drugs [4,64,65,66]. Researchers are also exploring the binding of other biologically active ligands using axially positioned tetravalent platinum groups, such as halogen, hydroxyl, and carboxyl. These investigations include the use of biologically targeted small molecules, non-steroidal anti-inflammatory drugs (NSAIDs), P53 agonists, alkylating agents, Histone deacetylase (HDAC) inhibitors, and targeted delivery molecules, among others (Figure 4) [21,67,68,69,70,71,72]. These ligands not only enhance the anti-tumor activity of platinum drugs by binding to them but also improve their physicochemical properties, such as transmembrane transporter rate, solubility, targeted delivery, and bioavailability, by increasing the lipid solubility of the compounds (Figure 5) [4,73].

### 3.1. Targeting p53-MDM2 Interaction with Pt(IV) Compounds

**Figure 6:**
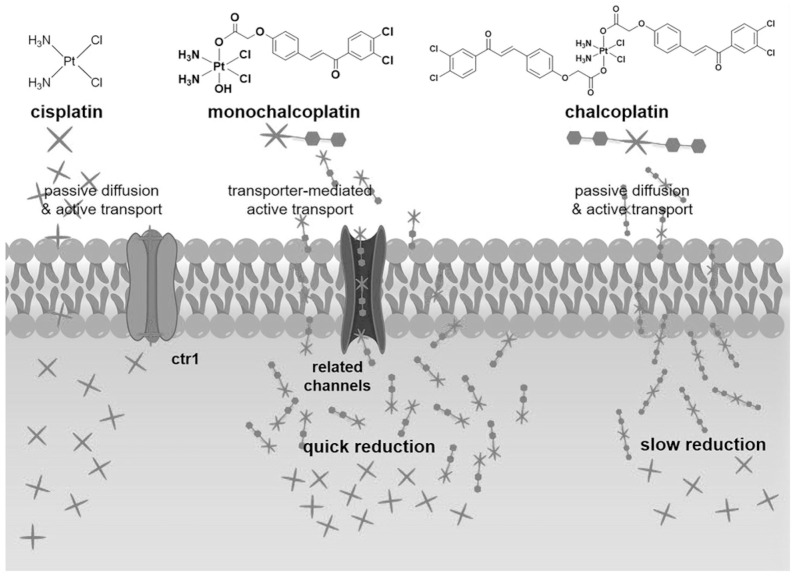
Cellular entry pathways and reduction processes of cisplatin, monochalcoplatin, and chalcoplatin. (Reprinted with permission from Ref. [68]. Copyright 2018 Wiley).

The multifunctional targeting compounds of Pt(IV) were initially designed to inhibit DNA damage repair, regulate the tumor microenvironment, enhance the sensitivity of tumor cells to platinum drugs, promote platinum drug transmembrane, and reduce the toxicity of platinum drugs to normal cells and tissues by targeting the abnormally expressed proteins of the tumor and DNA damage repair proteins [4,74]. For example, chalcone is an inhibitor of p53-MDM2 interaction. The activity of p53 is primarily regulated by MDM2, which encodes an E3 ubiquitin ligase. Aberrant amplification of MDM2 activity (including MDM2 gene amplification and overexpression) in a variety of tumors results in increased levels of MDM2 protein expression. Highly expressed MDM2 inhibits the function of the p53 protein by binding to and degrading p53, inhibiting cell cycle arrest and apoptosis, and attenuating its anti-cancer effects. Chalcone disrupts the binding of MDM2 to the p53 protein and restores the function of the tumor suppressor protein p53. Upon detection of DNA damage, the ‘restored’ p53 protein causes cell cycle arrest to ‘determine’ if the DNA damage can be effectively repaired; if not, p53 initiates programmed cell death, i.e., apoptosis. Guangyu Zhu’s team used 4-formylphenoxyacetic acid as an axial ligand and a building block for chalcone, which was reacted with tetravalent hydroxycisplatin or tetravalent hydroxyoxaliplatin [75]. The researchers designed and synthesized bifunctional Pt(IV) compounds called monochalcoplatin and chalcoplatin, which have monocarboxylated and dicarboxylated structures, respectively. It was observed that monochalcoplatin can be efficiently and rapidly taken up and accumulated by tumor cells, not solely due to its lipophilicity. Through platinum uptake and reduction assays, the researchers found that monochalcoplatin primarily enters cells through active transport rather than passive diffusion or phagocytosis/macrophagocytosis. The role of Ctr1 in the uptake of monochalcoplatin was determined to be insignificant (Figure 6). Further experimental data revealed that monochalcoplatin enters cells mainly through active transport and is rapidly released through reduction, causing DNA damage and apoptosis, ultimately exerting anti-tumor effects. Monochalcoplatin exhibited 422-fold greater anti-tumor activity than cisplatin, with IC_50_ values in the nanomolar range for both cisplatin-sensitive and resistant cells. In contrast, dicarboxylated Pt(IV) chalcoplatin showed anti-tumor activity at the micromolar level due to the slow reduction of chalcoplatin in tumor cells, which hindered the rapid induction of DNA lesions. Moreover, chalcoplatin, when combined with cisplatin and chalcone, increased the expression of p53 protein, demonstrating better anti-tumor activity than cisplatin in p53 wild-type cancer cells and effectively overcoming cisplatin resistance.

### 3.2. Targeting Cyclooxygenase-2 with Pt(IV) Compounds

**Figure 7:**
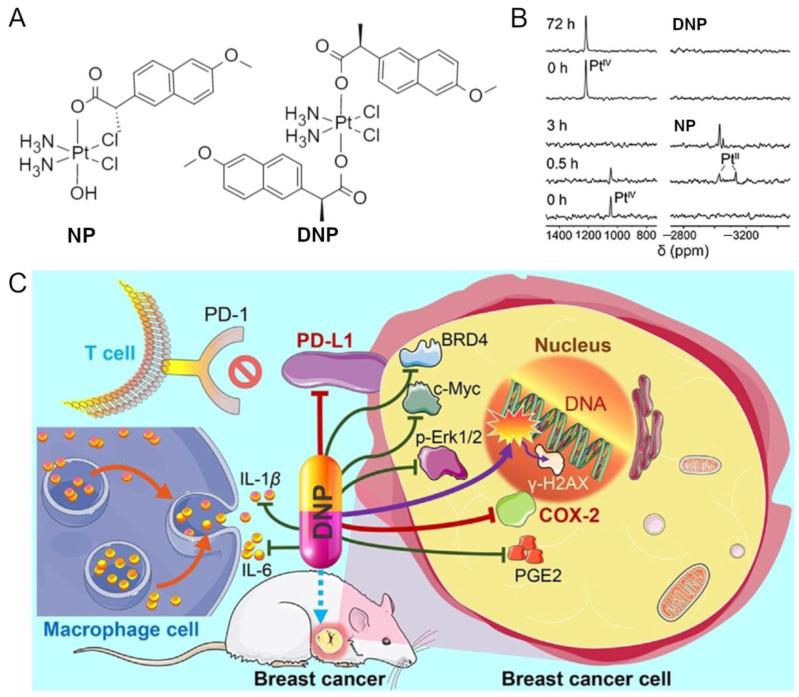
(**A**) Chemical structures of **DNP** and **NP**. (**B**) ^195^Pt-NMR spectroscopy for determining the amount of Pt(II) released by the reduction of **NP** and **DNP** under ASA incubation; (**C**) **DNP**’s putative anti-cancer mechanism. (Reprinted with permission from Ref. [37]. Copyright 2020 Wiley).

Cyclooxygenase-2 (COX-2) is closely associated with the expression of programmed death ligand 1 (PD-L1), which plays a significant role in the development of breast cancer. Overexpression of PD-L1 leads to immune evasion by cancer cells while blocking it stimulates anti-cancer immune responses. In a study conducted by Xiaoyong Wang’s team, two multispecific Pt(IV) complexes, namely **DNP** and **NP**, were prepared using the non-steroidal anti-inflammatory drug naproxen (NPX) as an axial ligand (Figure 7A) [37]. NPX is an active small molecule that inhibits the growth of breast cancer cells. Compared to cisplatin, **DNP** and **NP** showed significantly increased lipophilicity (Log P: **DNP**, 1.46; **NP**, 0.02 vs. cisplatin, Log P: −2.35). In the cellular platinum accumulation assay, after 24 h of incubation in MCF-7 breast cancer cells, **DNP** and **NP** exhibited intracellular platinum contents of 2.85 ng per microgram protein. This platinum content was 65- and 11-fold higher for **DNP** and **NP**, respectively, compared to CDDP and correlated positively with their lipophilicity. Additionally, **DNP** demonstrated better cytotoxicity than **NP** in the in vitro evaluation of anti-tumor bioactivity, showing comparable activity to other analogs or NSAID-platinum complexes [76,77,78]. When the extent of **DNP** reduction was examined in vivo, **DNP** remained unchanged after 72 h in the presence of both ASA and GSH, unlike **NP**, which was completely reduced after 3 h in ASA (Figure 7B). Further investigations into the anti-tumor mechanism of action revealed that **DNP** could downregulate the expression of COX-2 and PD-L1, inhibit prostaglandin secretion, reduce the expression of the breast cancer-related protein BRD4 and the phosphorylation of extracellular signal-regulated proteins 1/2 (ERK1/2), and block the expression of the breast cancer cell oncogene c-Myc (Figure 7C). Considering its extracellular anti-reducing properties, the researchers hypothesized that **DNP** could form chimeric adducts with cellular DNA. This unique characteristic allows **DNPs** to exert overall pharmacological activity, which is uncommon in the development of novel Pt(IV) derivatives.

### 3.3. Targeting Lipid Metabolism with Pt(IV) Compounds

In a separate study, researchers discovered that Pt(IV) compounds possess effective anti-tumor effects even without undergoing reduction. Changes in lipid metabolism are considered crucial indicators of cancer development [79,80]. In this study, the scientists utilized the lipid-lowering drug bezafibrate to modify cisplatin and successfully synthesized two novel Pt(IV) derivatives, **CB** and **CP**, employing a Pt(IV) design and synthesis strategy [38]. During the assessment of their in vitro anti-tumor activity, the Pt(IV) precursor drug **CB** demonstrated 187-fold higher anti-tumor activity in comparison to the clinical anti-cancer drug cisplatin while causing less harm to normal cells. To determine the rate of reduction release of the compounds, an in vitro reduction assay was conducted for **CB** and **CP** by adding 10 eq-treated ASA and analyzing the compounds using HPLC. The experimental data revealed that bezafibrate can be easily released from the monocarboxylated Pt(IV) complex **CB** in an in vitro reducing environment, resulting in significant synergistic anti-tumor activity. In contrast, even after 48 h of incubation with 10 eq-treated ASA, only less than 3% of the reduced dicarboxylated Pt(IV) compound **CP** was able to be reduced.

### 3.4. Novel Multifunctional Pt(IV) Dual-Prodrug Compounds

**Figure 8:**
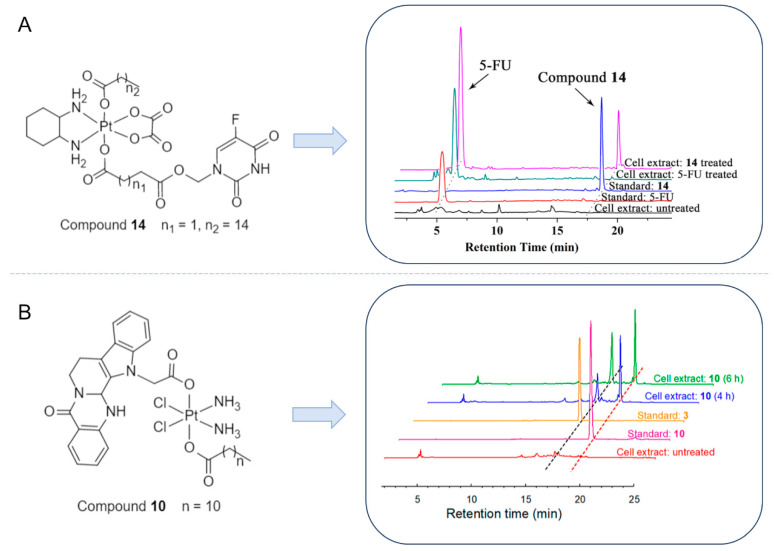
(**A**) The intracellular release of Pt(II) after incubation of compound **10** (100 μM) in MCF-7 cells at different times was determined by HPLC. (Reprinted with permission from Ref. [74]. Copyright 2023 American Chemical Society). (**B**) The intracellular release of Pt(II) after incubation of compound **14** (100 μM) in MCF-7 cells for different times was determined by HPLC. (Reprinted with permission from Ref. [75]. Copyright 2019 American Chemical Society).

In a study by Xu’s team [81], a dual pro-drug Pt(IV) was designed to enhance the anti-tumor effects of conventional platinum compounds with 5-fluorouracil (5-FU) in chemotherapy. The researchers aimed to improve the transmembrane uptake and lipid solubility of the pro-drug by introducing aliphatic hydrocarbon chains of varying lengths at the axial hydroxyl position. Among the synthesized ‘dual-prodrugs’, compound **14** demonstrated significantly higher intracellular platinum accumulation (62- and 825-fold compared to oxaliplatin and compound **8**, respectively) within 9 h. It also induced DNA damage, apoptosis, and inhibited cell migration and invasion in HCT-116 colorectal cancer cells. Moreover, compound **14** caused significant S and G2 phase cycle blockade and upregulated the expression of thymidine synthase and p53 in HCT-116 cells. In vivo evaluation in NOD/SCID mice revealed potent anti-tumor activity of compound **14** (tumor inhibition rate: 84.8%) with no significant toxicity, outperforming oxaliplatin (tumor inhibition rate: 57.8%). In the reductive release assay of compound **14**, the stability of the compound was first investigated. Compound **14** was found to be stable in the PBS/DMF (99:1) system for 48 h. To determine if the dual-prodrug **14** could release oxaliplatin and 5-FU intracellularly, the researchers obtained metabolic extracts of HCT-116 cells treated with the compounds. The extracts were then analyzed using high-performance liquid chromatography (HPLC) and high-resolution mass spectrometry (HRMS). After 4 h in the treated group, compound **14** was observed to readily release 5-FU under intracellular conditions, as analyzed above (Figure 8A). This indicates that compound **14** exerts its anti-tumor effect through a synergistic action of divalent platinum and 5-FU. In another study conducted by Xu’s team [82], they designed and synthesized multi-targeted Evodiamine-Pt(IV) anti-cancer pro-drugs (compounds **4**–**14**) using plant-derived Evodiamine (EVO) and conventional Pt(II) drugs as starting materials. Among these compounds, compound **10** exhibited in vitro anti-tumor activity 118-fold higher than that of cisplatin while also showing lower toxicity to normal cells. Further investigations revealed that compound **10** significantly increased intracellular platinum accumulation and DNA damage, disrupted mitochondrial membrane potential, inhibited cell migration and invasion, upregulated intracellular reactive oxygen species levels, and induced apoptosis and autophagic cell death. Similar experimental results were obtained for compound **14** in the reductive release assay (Figure 8B).

## 4. Effect of Different Reducing Agents on the Reductive Release Rate of Pt(IV) Compounds

**Figure 9:**
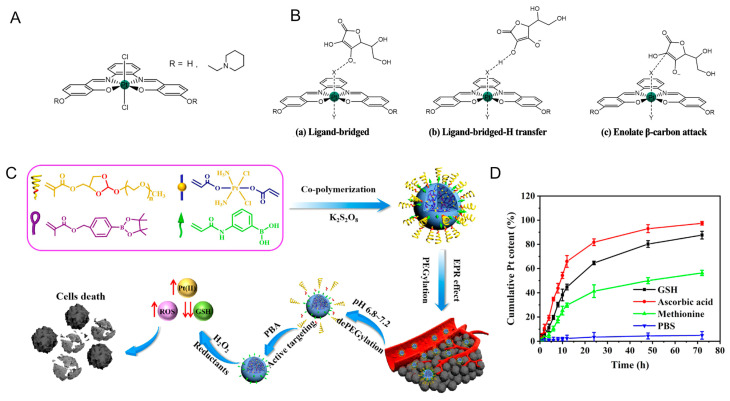
(**A**) Chemical structure of **Pt(IV)-Sal**. (**B**) Mechanisms for reducing Pt(IV) complexes by AsA^−^ and Cys^−^ in the inner sphere. (Reprinted with permission from Ref. [76]. Copyright 2022 MDPI). (**C**) Synthesis route of Pt(IV)-cross-linked nanogels and the delivery mechanism of action of the complexes. (**D**) Pt(II) release from Pt(IV) after in vitro incubation with different reducing agents (Reprinted with permission from Ref. [19]. Copyright 2021 Elsevier).

The study investigated the ability of bioreducing agents such as AsA and GSH to convert octahedral **Pt(IV)-Sal** precursors into square-planar **Pt(II)-Sal** G-Q conjugates using quantum mechanical density functional theory (DFT) calculations (Figure 9A) [83]. The key step in the mechanism of action of the Pt(IV) precursors is the intracellular reduction, which leads to the release of the corresponding Pt(II) ligand and axial molecules from the Pt(IV) compound. The reduction mechanisms of Pt(IV) complexes are generally classified into inner- and outer-sphere [83,84,85,86]. The former involves direct interactions between the reacting species, forming new bonds, while the latter involves the transfer of electrons without direct interactions. As mentioned in Dan Gibson’s review, most studies on the reduction of Pt(IV) complexes containing chlorine or hydroxyl ligands by ascorbic acid have shown that the reduction mechanism is either an outer-sphere mechanism or an inner-sphere mechanism catalyzed by Pt(II) [36]. In the presence of AsA and GSH bioreductants, researchers identified three possible inner-sphere mechanisms: (a) ligand-bridged, (b) ligand-bridged H-transfer, and (c) enolated β-carbon attack (Figure 9B) [83]. Mechanism (A) coexists with AsA and GSH, while mechanisms (b) and (c) operate when only AsA acts as a reducing agent. Additionally, the active form of AsA is the monoanionic form (AsA^−^), which is most abundant at physiological pH (pKa around 3.8). L-cysteine (Cys) has been used as a model for sulfur-containing bioreductants. Figure 9B illustrates that in both mechanisms (a) and (b), one of the axial ligands of the Pt(IV) compounds forms a bridge between the platinum center and the reducing agent, facilitating the flow of electrons. However, in mechanism (b), electron transfer is accompanied by proton transfer from the reducing agent to the ligand. The choice between mechanisms (a) and (b) depends on the homogeneity of the axial ligand. For instance, unstable ligands like halides follow mechanism (a), while the release of less stable ligands such as hydroxides and carboxylates is promoted by proton transfer, as in mechanism (b). Mechanism (c) involves nucleophilic attack of the alkene β-carbon on the axial ligand [87], forming a new bond with the carbon atom and subsequent detachment.

The chemotherapeutic efficacy of Pt(II) is often hindered by low intracellular drug concentration and GSH-mediated detoxification. To address these challenges, researchers developed a nanogel (~160 nm) through copolymerization of four functional monomers (Figure 9C) [19]. The hydrophilic methoxypolyethylene glycols (Mpeg) were randomly distributed on the surface of the nanoparticles, rendering the nanogel invisible in the bloodstream. Chemical cross-linking of Pt(IV) linkers inside the particles significantly enhanced the in vivo stability of the nanogel. Importantly, this nanogel could effectively deplete intracellular GSH through dual modulation of Pt(IV) and arylboronic acid esters, thereby increasing the toxicity of Pt(II) both in vivo and in vitro. In the in vivo evaluation of the compound’s activity, the platinum nanocompound exhibited a tumor growth inhibition rate (TGI) of 79.14% in A549/CDDP-resistant nude mice, compared to the Pt(II) compound (TGI: 17.27%). The stability and reductive release properties of this compound were also assessed. To simulate the intracellular reductive environment, the drug was released in vitro using endogenous reducing agents such as ASA, GSH, and methionine. In the absence of reducing agents, less than 5% of Pt(II) was released within 72 h in a pH 7.4 solution, indicating that the nanogel effectively maintained the drug stability through chemical cross-linking agents. However, the addition of a 10 mM reducing agent significantly increased the release rate due to the reducing effect. Furthermore, there were notable differences in the assay results among the three reducing agents. Notably, over 80% of ascorbic acid was released within 24 h, surpassing the release of glutathione and methionine (Figure 9D). This discrepancy can be attributed to variations in the ionized forms and the reaction mechanism of the reducing agents [88,89,90].

In a study of racemic platinum, a tetravalent platinum compound, Raynaud’s team found that the composition and proportion of biotransformation products in the ultrafiltration fraction of cells were influenced by intracellular GSH levels [91,92]. They also discovered that one of these biotransformation products contained GSH adducts. Similarly, Nakai reported that cisplatin-GSH adducts are formed when cisplatin-tetrachloro-Pt(IV) complexes are reduced by GSH, effectively sequestering the cisplatin from the DNA [93]. Further investigation is required to determine if the biotransformation product of the Pt(IV) compound, which is not reduced in the presence of a reducing agent, is associated with a spatial site resistance that prevents the formation of adducts with GSH. Additionally, it has been demonstrated that NADH (as a source of reducing equivalents) and Cytc play a crucial role in facilitating the reduction of Pt(IV) to Pt(II) [94].

## 5. Other Multifunctional Targeted Pt(IV) Compounds

**Figure 10:**
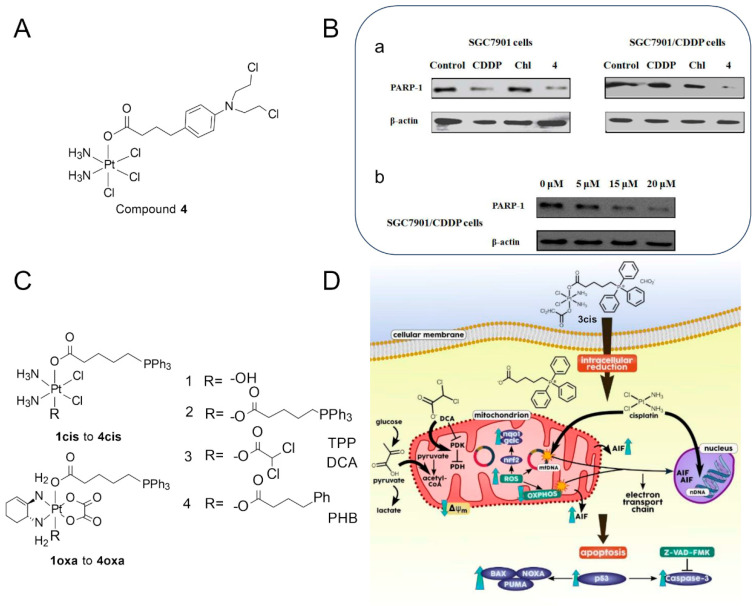
(**A**) Chemical structure of compound **4**. (**B**) (**a**) The expression of PARP-1 was measured at different compounds. (**b**) Inhibition of PARP-1 expression was tested by administering compound **4** at different concentrations. (Reprinted with permission from Ref. [90]. Copyright 2017 Elsevier). All rights reserved. (**C**) Chemical structures of mitochondria-targeting bifunctional Pt(IV) pro-drugs. (**D**) The mechanism of action diagram of **3cis**. (Reprinted with permission from Ref. [91]. Copyright 2019 Wiley).

After platinum compounds damage tumor cell DNA, the DNA damage stress response (DDR) is initiated to recruit DNA damage-related proteins for repair. The repair pathways for DNA, which include Nucleotide Excision Repair (NER), Base Excision Repair (BER), Homologous Recombination (HR), Non-homologous end joining (NHEJ), and Fanconi anemia (FA) pathway mechanisms, primarily involve ICL repair [95,96]. Proteins such as PARP, RPA, Rad51, ERCC1-XPF, and the FANC family collaborate in the repair of damaged DNA [95,97]. In this study, researchers designed and prepared two Pt(IV) complexes by combining cisplatin and platinum oxalate units with the DNA-damaging agent chlorobenzenebutyronitrile (Figure 10A). The design was based on targeting inhibitors of the DNA damage repair protein PARP. By enhancing DNA damage through this combined action, the conjugates exhibited anti-tumor activity comparable to that of cisplatin and oxaliplatin on all tested cancer cells and, to some extent, overcome cisplatin resistance. Unlike cisplatin and oxaliplatin, the mixture of cisplatin and chlorambucil **4** arrested the cell cycle in the S and G2 phases. Apoptosis studies demonstrated that compound **4** induced significant apoptosis in both SGC7901 and SGC7901/CDDP cells. Furthermore, further investigations revealed that compound **4** overcame resistance by promoting platinum uptake and inhibiting PARP-1 protein expression (Figure 10B) [98]. These findings suggest that the strategy of ‘combined action’ of DNA is an effective approach to overcoming cisplatin resistance. In another study, researchers conducted experiments to develop new mitochondria-targeted Pt(IV) complexes (**1cis**-**4cis**, **1oxa**-**4oxa**) with DCA or PHB ligands. The aim was to increase the accumulation of these complexes within the mitochondria, thereby enhancing the anti-tumor effect of PDK inhibitors combined with platinum compounds (Figure 10C) [99]. The results of in vitro activity assays revealed that the platinum complexes aggregated in both the mitochondria and nuclei, resulting in a synergistic interaction between the platinum center of **3cis** and the organic mitochondrial sensitizers. This led to a significant improvement in in vivo activity, as evidenced by a more than 5-fold reduction in tumor volume compared to CDDP. Furthermore, the researchers developed **3cis** liposomes to target tumors at an even higher level, which resulted in complete tumor remission and increased anti-cancer activity. Additionally, the researchers demonstrated that coupling DCA to the center of Pt(IV) prevented kidney injury induced by platinum and reduced the nephrotoxic side effects of Pt(II) (Figure 10D).

## 6. Conclusions and Perspectives

In certain cases, Pt(IV) compounds enter the cell and interact with DNA in the form of hydrates, following mono- or bi-hydrolysis of the leaving group in the platinum equatorial plane. The rate of hydrolysis is influenced by factors such as spatial site resistance, pH, and pKa [3,36]. When Pt(IV) compounds cross-link with DNA, they primarily form interstrand cross-linking (ICL) adducts. These cross-linking products have been observed to interact rapidly and directly with DNA, with the platinum compounds maintaining their Pt(IV) state in the products (Figure 5) [40]. Similar cross-linking results have been observed for other tetravalent platinum compounds like oxaliplatin and satraplatin [40]. Compared to intrastrand cross-linking, ICLs obstruct essential DNA metabolic pathways (e.g., replication, transcription, and metabolism), leading to the generation of more cytotoxic DNA damage [100,101]. The formation of rapid and stable DNA interstrand cross-linking products may be a crucial mechanism for the functioning of Pt(IV) compounds. Additionally, Pt(IV) compounds exert a holistic effect by influencing the expression of key intracellular proteins, including immune inhibitory molecules (PD-L1), tumor inflammatory factors (IL-1 and IL-6), cyclooxygenase (COX-2), and oncogenes (c-MYC). Although the precise mechanism underlying the anti-tumor activity of Pt(IV) compounds is not fully understood, exploring this holistic molecular approach to anti-tumor activity is of great interest. Moreover, investigating whether Pt(II) drugs can be activated through photodynamic and electrochemical methods for targeted photo-activation of Pt(IV) drugs without compromising healthy tissues presents a challenging task in cases where the anti-tumor activity of Pt(IV) compounds is hindered due to limited in vivo reduction and release [102].

Several Pt(IV) compounds have been investigated in both preclinical and clinical trials, although their widespread clinical use is still limited. However, initial clinical studies have indicated that Pt(IV) compounds demonstrate improved anti-tumor activity and lower toxicity in specific types of cancer. Nevertheless, further clinical trials and rigorous studies are required to validate their safety and efficacy. Moreover, despite the potential advantages and application prospects of Pt(IV) compounds, there are existing limitations in drug discovery and development. It is crucial to consider the form and transporter protein responsible for facilitating the entry of Pt(IV) compounds into cells to exert their effects. Firstly, there is a relative lack of research on the pharmacokinetics, metabolism, and catabolism mechanism of Pt(IV) compounds, which needs to be addressed. Gaining a deeper understanding of these properties will aid in optimizing drug design and usage. Secondly, the heterogeneity of Pt(IV) compounds poses a challenge for their clinical applications. Variations in chemical properties and ligand designs can affect their stability and activity. Furthermore, the reusability of these compounds requires further investigation. Finally, it is crucial for researchers to better comprehend the side effects and toxicity of Pt(IV) compounds in order to effectively manage and mitigate any adverse effects faced by patients. Although there are challenges and shortcomings, Pt(IV) compounds show promise as pro-drugs for platinum-based anti-tumor research. Enhancements in targeting strategies, combination therapies, new synthetic methods, and combinatorial chemistry strategies can improve the design and development of Pt(IV) compounds. With the advancement of clinical applications and drug development, multifunctional targeted Pt(IV) compounds are anticipated to emerge as significant drug candidates for cancer therapy in the future.

## Data Availability

Not applicable.

## References

[1] Khoury A., Deo K.M., Aldrich-Wright J.R. (2020). Recent advances in platinum-based chemotherapeutics that exhibit inhibitory and targeted mechanisms of action. J. Inorg. Biochem..

[2] Pan Z., Zheng J., Zhang J., Lin J., Lai J., Lyu Z., Feng H., Wang J., Wu D., Li Y. (2022). A Novel Protein Encoded by Exosomal CircATG4B Induces Oxaliplatin Resistance in Colorectal Cancer by Promoting Autophagy. Adv. Sci..

[3] Wilson J.J., Lippard S.J. (2013). Synthetic Methods for the Preparation of Platinum Anti-cancer Complexes. Chem. Rev..

[4] Johnstone T.C., Suntharalingam K., Lippard S.J. (2016). The Next Generation of Platinum Drugs: Targeted Pt(II) Agents, Nanoparticle Delivery, and Pt(IV) Pro-drugs. Chem. Rev..

[5] da Costa A.A.B.A., Baiocchi G. (2021). Genomic profiling of platinum-resistant ovarian cancer: The road into druggable targets. Semin. Cancer Biol..

[6] Gibson D. (2019). Multi-action Pt(IV) anti-cancer agents; do we understand how they work?. J. Inorg. Biochem..

[7] Reed E. (1998). Platinum-DNA adduct, nucleotide excision repair and platinum based anti-cancer chemotherapy. Cancer Treat. Rev..

[8] Wang D., Lippard S.J. (2005). Cellular processing of platinum anti-cancer drugs. Nat. Rev. Drug Discov..

[9] Dasari S., Njiki S., Mbemi A., Yedjou C.G., Tchounwou P.B. (2022). Pharmacological Effects of Cisplatin Combination with Natural Products in Cancer Chemotherapy. Int. J. Mol. Sci..

[10] Galluzzi L., Senovilla L., Vitale I., Michels J., Martins I., Kepp O., Castedo M., Kroemer G. (2012). Molecular mechanisms of cisplatin resistance. Oncogene.

[11] Kleih M., Böpple K., Dong M., Gaißler A., Heine S., Olayioye M.A., Aulitzky W.E., Essmann F. (2019). Direct impact of cisplatin on mitochondria induces ROS production that dictates cell fate of ovarian cancer cells. Cell Death Dis..

[12] Oun R., Moussa Y.E., Wheate N.J. (2018). The side effects of platinum-based chemotherapy drugs: A review for chemists. Dalton Trans..

[13] Tanase D.M., Gosav E.M., Radu S., Costea C.F., Ciocoiu M., Carauleanu A., Lacatusu C.M., Maranduca M.A., Floria M., Rezus C. (2019). The Predictive Role of the Biomarker Kidney Molecule-1 (KIM-1) in Acute Kidney Injury (AKI) Cisplatin-Induced Nephrotoxicity. Int. J. Mol. Sci..

[14] Zheng Y., Deng Z., Tang M., Xiao D., Cai P. (2020). Impact of genetic factors on platinum-induced gastrointestinal toxicity. Mutat. Res. Rev. Mutat. Res..

[15] Canil G., Braccini S., Marzo T., Marchetti L., Pratesi A., Biver T., Funaioli T., Chiellini F., Hoeschele J.D., Gabbiani C. (2019). Photocytotoxic Pt(iv) complexes as prospective anti-cancer agents. Dalton Trans..

[16] Fu Y., Kong Y., Li X., Cheng D., Hou Y., Li Y., Li T., Xiao Y., Zhang Q., Rong R. (2023). Novel Pt(IV) pro-drug self-assembled nanoparticles with enhanced blood circulation stability and improved anti-tumor capacity of oxaliplatin for cancer therapy. Drug Deliv..

[17] Ma L., Lin X., Li C., Xu Z., Chan C.Y., Tse M.K., Shi P., Zhu G. (2018). A Cancer Cell-Selective and Low-Toxic Bifunctional Heterodinuclear Pt(IV)–Ru(II) Anti-cancer Pro-drug. Inorg. Chem..

[18] Yang L., Yan G., Wang S., Xu J., Fang Q., Xue Y., Yang L., Xu X., Tang R. (2021). Dynamic precise dual-drug-backboned nano-prodrugs for selective chemotherapy. Acta Biomater..

[19] Xu J., Hu T., Zhang M., Feng P., Wang X., Cheng X., Tang R. (2021). A sequentially responsive nanogel via Pt(IV) cross-linking for overcoming GSH-mediated platinum resistance. J. Colloid Interface Sci..

[20] Arnesano F., Natile G. (2021). Interference between copper transport systems and platinum drugs. Semin. Cancer Biol..

[21] Spector D., Erofeev A., Gorelkin P., Skvortsov D., Trigub A., Markova A., Nikitina V., Ul’Yanovskiy N., Shtil’ A., Semkina A. (2023). Biotinylated Pt(iv) pro-drugs with elevated lipophilicity and cytotoxicity. Dalton Trans..

[22] van der Vijgh W.J. (1991). Clinical Pharmacokinetics of Carboplatin. Clin. Pharmacokinet..

[23] Han X., Sun J., Wang Y., He Z. (2015). Recent Advances in Platinum (IV) Complex-Based Delivery Systems to Improve Platinum (II) Anti-cancer Therapy. Med. Res. Rev..

[24] Choroba K., Machura B., Szlapa-Kula A., Malecki J.G., Raposo L., Roma-Rodrigues C., Cordeiro S., Baptista P.V., Fernandes A.R. (2021). Square planar Au(III), Pt(II) and Cu(II) complexes with quinoline-substituted 2,2′:6′,2″-terpyridine ligands: From in vitro to in vivo biological properties. Eur. J. Med. Chem..

[25] Date T., Kuche K., Ghadi R., Kumar P., Jain S. (2022). Understanding the Role of Axial Ligands in Modulating the Biopharmaceutical Outcomes of Cisplatin(IV) Derivatives. Mol. Pharm..

[26] Lucaciu R.L., Hangan A.C., Sevastre B., Oprean L.S. (2022). Metallo-Drugs in Cancer Therapy: Past, Present and Future. Molecules.

[27] Sun Y., Lu Y., Bian M., Yang Z., Ma X., Liu W. (2021). Pt(II) and Au(III) complexes containing Schiff-base ligands: A promising source for anti-tumor treatment. Eur. J. Med. Chem..

[28] Bai L., Gao C., Liu Q., Yu C., Zhang Z., Cai L., Yang B., Qian Y., Yang J., Liao X. (2017). Research progress in modern structure of platinum complexes. Eur. J. Med. Chem..

[29] Rezaee M., Sanche L., Hunting D.J. (2013). Cisplatin Enhances the Formation of DNA Single- and Double-Strand Breaks by Hydrated Electrons and Hydroxyl Radicals. Radiat. Res..

[30] Sava G., Jaouen G., Hillard E.A., Bergamo A. (2012). Targeted therapy vs. DNA-adduct formation-guided design: Thoughts about the future of metal-based anti-cancer drugs. Dalton Trans..

[31] Boldinova E.O., Yudkina A.V., Shilkin E.S., Gagarinskaya D.I., Baranovskiy A.G., Tahirov T.H., Zharkov D.O., Makarova A.V. (2021). Translesion activity of PrimPol on DNA with cisplatin and DNA–protein cross-links. Sci. Rep..

[32] Ang W.H., Myint M., Lippard S.J. (2010). Transcription Inhibition by Platinum-DNA Cross-Links in Live Mammalian Cells. J. Am. Chem. Soc..

[33] Todd R.C., Lippard S.J. (2010). Structure of duplex DNA containing the cisplatin 1,2-{Pt(NH_3_)_2_}^2+^-d(GpG) cross-link at 1.77 Å resolution. J. Inorg. Biochem..

[34] Wang D., Zhu G., Huang X., Lippard S.J. (2010). X-ray structure and mechanism of RNA polymerase II stalled at an antineoplastic monofunctional platinum-DNA adduct. Proc. Natl. Acad. Sci. USA.

[35] Ponte F., Scoditti S., Mazzone G., Sicilia E. (2023). The current status in computational exploration of Pt(iv) pro-drug activation by reduction. Phys. Chem. Chem. Phys..

[36] Wexselblatt E., Gibson D. (2012). What do we know about the reduction of Pt(IV) pro-drugs?. J. Inorg. Biochem..

[37] Jin S., Muhammad N., Sun Y., Tan Y., Yuan H., Song D., Guo Z., Wang X. (2020). Multispecific Platinum(IV) Complex Deters Breast Cancer via Interposing Inflammation and Immunosuppression as an Inhibitor of COX-2 and PD-L1. Angew. Chem. Int. Ed..

[38] Qiao X., Gao Y.Y., Zheng L.X., Ding X.J., Xu L.W., Hu J.J., Gao W.Z., Xu J.Y. (2021). Targeting ROS-AMPK pathway by multiaction Platinum(IV) pro-drugs containing hypolipidemic drug bezafibrate. Eur. J. Med. Chem..

[39] Song X.Q., Liu R.P., Wang S.Q., Li Z., Ma Z.Y., Zhang R., Xie C.Z., Qiao X., Xu J.Y. (2020). Anti-cancer Melatplatin Pro-drugs: High Effect and Low Toxicity, MT1-ER-Target and Immune Response In Vivo. J. Med. Chem..

[40] Zhao C.L., Qiao X., Liu X.M., Song X.Q., Zou Y.H., Li D.Q., Yu X.W., Bao W.G., Xu J.Y. (2022). Rapid DNA interstrand cross-linking of Pt(IV) compound. Eur. J. Pharmacol..

[41] Chang C.-H., Qiu J., O’Sullivan D., Buck M.D., Noguchi T., Curtis J.D., Chen Q., Gindin M., Gubin M.M., van der Windt G.J.W. (2015). Metabolic Competition in the Tumor Microenvironment Is a Driver of Cancer Progression. Cell.

[42] Berger I., Nazarov A.A., Hartinger C.G., Groessl M., Valiahdi S.M., Jakupec M.A., Keppler B.K. (2007). A glucose derivative as natural alternative to the cyclohexane-1,2-diamine ligand in the anti-cancer drug oxaliplatin?. ChemMedChem.

[43] Zhang Q., Shao J., Wang J., Gong X.-J., Liu W.-X., Wang S., Zhang Y., Yang S., Zhang Q.-S., Wei J.-X. (2022). Anti-tumor effects of new glycoconjugated PtII agents dual-targeting GLUT1 and Pgp proteins. Dalton Trans..

[44] Criado J.J., Garcia-Moreno M., Macias R.R., Marin J.J., Medarde M., Rodriguez-Fernandez E. (1999). Synthesis and characterization of Sodium cis-dichlorochenodeoxycholylglycinato(O,N) platinum(II)—Cytostatic activity. BioMetals.

[45] Criado J.J., Manzano J.L., Rodríguez-Fernández E. (2003). New organotropic compounds. J. Inorg. Biochem..

[46] Trauner M., Boyer J.L. (2003). Bile Salt Transporters: Molecular Characterization, Function, and Regulation. Physiol. Rev..

[47] Barbara C., Orlandi P., Bocci G., Fioravanti A., Di Paolo A., Natale G., Del Tacca M., Danesi R. (2006). In vitro and in vivo antitumour effects of novel, orally active bile acid-conjugated platinum complexes on rat hepatoma. Eur. J. Pharmacol..

[48] Seroka B., Łotowski Z., Hryniewicka A., Rárová L., Sicinski R.R., Tomkiel A.M., Morzycki J.W. (2020). Synthesis of New Cisplatin Derivatives from Bile Acids. Molecules.

[49] Nakhaei E., Kim C.W., Funamoto D., Sato H., Nakamura Y., Kishimura A., Mori T., Katayama Y. (2017). Design of a ligand for cancer imaging with long blood circulation and an enhanced accumulation ability in tumors. MedChemComm.

[50] Weitman S.D., Lark R.H., Coney L.R., Fort D.W., Frasca V., Zurawski V.R., Kamen B.A. (1992). Distribution of the Folate Receptor GP38 in Normal and Malignant Cell Lines and Tissues. Cancer Res..

[51] Jennifer-Sudimack B.A., Lee R.J. (2002). Targeted drug delivery via the folate receptor. Adv. Drug Deliv. Rev..

[52] Morales-Cruz M., Cruz-Montañez A., Figueroa C.M., González-Robles T., Davila J., Inyushin M., Loza-Rosas S.A., Molina A.M., Muñoz-Perez L., Kucheryavykh L.Y. (2016). Combining Stimulus-Triggered Release and Active Targeting Strategies Improves Cytotoxicity of Cytochrome c Nanoparticles in Tumor Cells. Mol. Pharm..

[53] Nahire R., Haldar M.K., Paul S., Ambre A.H., Meghnani V., Layek B., Katti K.S., Gange K.N., Singh J., Sarkar K. (2014). Multifunctional polymersomes for cytosolic delivery of gemcitabine and doxorubicin to cancer cells. Biomaterials.

[54] Wen Y., Graybill W.S., Previs R.A., Hu W., Ivan C., Mangala L.S., Zand B., Nick A.M., Jennings N.B., Dalton H.J. (2015). Immunotherapy Targeting Folate Receptor Induces Cell Death Associated with Autophagy in Ovarian Cancer. Clin. Cancer Res..

[55] Vitols K., Montejano Y., Duffy T., Pope L., Grundler G., Huennekens F. (1987). Platinum-Folate Compounds: Synthesis, Properties and Biological Activity. Adv. Enzyme Regul..

[56] Pierroz V., Joshi T., Leonidova A., Mari C., Schur J., Ott I., Spiccia L., Ferrari S., Gasser G. (2012). Molecular and Cellular Characterization of the Biological Effects of Ruthenium(II) Complexes Incorporating 2-Pyridyl-2-pyrimidine-4-carboxylic Acid. J. Am. Chem. Soc..

[57] Li J., He X., Zou Y., Chen D., Yang L., Rao J., Chen H., Chan M.C.W., Li L., Guo Z. (2017). Mitochondria-targeted platinum(ii) complexes: Dual inhibitory activities on tumor cell proliferation and migration/invasion via intracellular trafficking of β-catenin. Metallomics.

[58] Qin Q.P., Wang Z.F., Huang X.L., Tan M.X., Luo Z.H., Wang S.L., Zou B.Q., Liang H. (2019). Two telomerase-targeting Pt(ii) complexes of jatrorrhizine and berberine derivatives induce apoptosis in human bladder tumor cells. Dalton Trans..

[59] Chao H., Liu J.-G., Jiang C.-W., Ji L.-N., Li X.-Y., Feng C.-L. (2001). Stereoisomerically controlled supramolecular architectures: A new strategy for the construction of enantio- and diastereomerically pure multinuclear RuII complexes. Inorg. Chem. Commun..

[60] Xiong K., Ouyang C., Liu J., Karges J., Lin X., Chen X., Chen Y., Wan J., Ji L., Chao H. (2022). Chiral RuII-PtIIComplexes Inducing Telomere Dysfunction against Cisplatin-Resistant Cancer Cells. Angew. Chem. Int. Ed..

[61] Bhargava A., Vaishampayan U.N. (2009). Satraplatin: Leading the new generation of oral platinum agents. Expert Opin. Investig. Drugs.

[62] Choy H., Park C., Yao M. (2008). Current Status and Future Prospects for Satraplatin, an Oral Platinum Analogue. Clin. Cancer Res..

[63] Doshi G., Sonpavde G., Sternberg C.N. (2012). Clinical and pharmacokinetic evaluation of satraplatin. Expert Opin. Drug Metab. Toxicol..

[64] Dong J., Tian H., Song C., Shi T., Elding L.I. (2018). Reduction of ormaplatin by an extended series of thiols unravels a remarkable correlation. Dalton Trans..

[65] Rischin D., Ling V. (1996). Ormaplatin resistance is associated with decreased accumulation of its platinum (II) analogue, dichloro(D,L-trans)1,2-diaminocyclohexaneplatinum(II). Br. J. Cancer.

[66] Trask C., Silverstone A., Ash C.M., Earl H., Irwin C., Bakker A., Tobias J.S., Souhami R.L. (1991). A Randomized Trial of Carboplatin Versus Iproplatin in Untreated Advanced Ovarian Cancer. J. Clin. Oncol..

[67] Li X., Liu Y., Tian H. (2018). Current Developments in Pt(IV) Pro-drugs Conjugated with Bioactive Ligands. Bioinorg. Chem. Appl..

[68] Mu M., Zhan J., Dai X., Gao H. (2022). Research progress of azido-containing Pt(IV) anti-tumor compounds. Eur. J. Med. Chem..

[69] Spector D., Krasnovskaya O., Pavlov K., Erofeev A., Gorelkin P., Beloglazkina E., Majouga A. (2021). Pt(IV) Pro-drugs with NSAIDs as Axial Ligands. Int. J. Mol. Sci..

[70] Spector D., Pavlov K., Beloglazkina E., Krasnovskaya O. (2022). Recent Advances in Light-Controlled Activation of Pt(IV) Pro-drugs. Int. J. Mol. Sci..

[71] Siddik Z., Hagopian G., Thai G., Tomisaki S., Toyomasu T., Khokhar A. (1999). Role of p53 in the ability of 1,2-diaminocyclohexane-diacetato-dichloro-Pt(IV) to circumvent cisplatin resistance. J. Inorg. Biochem..

[72] Zheng S., Li G., Shi J., Liu X., Li M., He Z., Tian C., Kamei K.I. (2023). Emerging platinum(IV) pro-drug nanotherapeutics: A new epoch for platinum-based cancer therapy. J. Control. Release.

[73] Ravera M., Gabano E., McGlinchey M.J., Osella D. (2022). Pt(iv) anti-tumor pro-drugs: Dogmas, paradigms, and realities. Dalton Trans..

[74] Tsvetkova D., Ivanova S. (2022). Application of Approved Cisplatin Derivatives in Combination Therapy against Different Cancer Diseases. Molecules.

[75] Ma L., Wang N., Ma R., Li C., Xu Z., Tse M.K., Zhu G. (2018). Monochalcoplatin: An Actively Transported, Quickly Reducible, and Highly Potent PtIV Anti-cancer Pro-drug. Angew. Chem. Int. Ed. Engl..

[76] Chen Y., Wang Q., Li Z., Liu Z., Zhao Y., Zhang J., Liu M., Wang Z., Li D., Han J. (2020). Naproxen platinum(iv) hybrids inhibiting cycloxygenases and matrix metalloproteinases and causing DNA damage: Synthesis and biological evaluation as anti-tumor agents in vitro and in vivo. Dalton Trans..

[77] Pathak R.K., Marrache S., Choi J.H., Berding T.B., Dhar S. (2014). The Pro-drug Platin-A: Simultaneous Release of Cisplatin and Aspirin. Angew. Chem. Int. Ed. Engl..

[78] Song X.Q., Ma Z.Y., Wu Y.G., Dai M.L., Wang D.B., Xu J.Y., Liu Y. (2019). New NSAID-Pt(IV) pro-drugs to suppress metastasis and invasion of tumor cells and enhance anti-tumor effect in vitro and in vivo. Eur. J. Med. Chem..

[79] Białek A., Jelińska M., Białek M., Lepionka T., Czerwonka M., Czauderna M. (2020). The Effect of Diet Supplementation with Pomegranate and Bitter Melon on Lipidomic Profile of Serum and Cancerous Tissues of Rats with Mammary Tumours. Antioxidants.

[80] Valentín-Guillama G., López S., Kucheryavykh Y.V., Chorna N.E., Pérez J., Ortiz-Rivera J., Inyushin M., Makarov V., Valentín-Acevedo A., Quinones-Hinojosa A. (2018). HIV-1 Envelope Protein gp120 Promotes Proliferation and the Activation of Glycolysis in Glioma Cell. Cancers.

[81] Zhang R., Song X.Q., Liu R.P., Ma Z.Y., Xu J.Y. (2019). Fuplatin: An Efficient and Low-Toxic Dual-Prodrug. J. Med. Chem..

[82] Liu X.M., Li Z., Xie X.R., Wang J.Q., Qiao X., Qiao X., Xie C.Z., Xu J.Y. (2023). Combination of DNA Damage, Autophagy, and ERK Inhibition: Novel Evodiamine-Inspired Multi-Action Pt(IV) Pro-drugs with High-Efficiency and Low-Toxicity Anti-tumor Activity. J. Med. Chem..

[83] Vigna V., Scoditti S., Spinello A., Mazzone G., Sicilia E. (2022). Anti-cancer Activity, Reduction Mechanism and G-Quadruplex DNA Binding of a Redox-Activated Platinum(IV)–Salphen Complex. Int. J. Mol. Sci..

[84] Bodappa N. (2020). Rapid assessment of platinum disk ultramicroelectrodes’ sealing quality by a cyclic voltammetry approach. Anal. Methods.

[85] Ponte F., Russo N., Sicilia E. (2018). Insights from Computations on the Mechanism of Reduction by Ascorbic Acid of PtIV Pro-drugs with Asplatin and Its Chlorido and Bromido Analogues as Model Systems. Chem.-Eur. J..

[86] Tanimoto S., Ichimura A. (2013). Discrimination of Inner- and Outer-Sphere Electrode Reactions by Cyclic Voltammetry Experiments. J. Chem. Educ..

[87] Ejehi Z., Ariafard A. (2017). A computational mechanistic investigation into the reduction of Pt(iv) pro-drugs with two axial chlorides by biological reductants. Chem. Comm..

[88] Dong J., Ren Y., Huo S., Shen S., Xu J., Tian H., Shi T. (2016). Reduction of ormaplatin and cis-diamminetetrachloroplatinum(iv) by ascorbic acid and dominant thiols in human plasma: Kinetic and mechanistic analyses. Dalton Trans..

[89] Lemma K., Berglund J., Farrell N., Elding L.I. (2000). Kinetics and mechanism for reduction of anticancer-active tetrachloroam(m)ine platinum(IV) compounds by glutathione. J. Biol. Inorg. Chem..

[90] Weaver E.L., Bose R.N. (2003). Platinum(II) catalysis and radical intervention in reductions of platinum(IV) anti-tumor drugs by ascorbic acid. J. Inorg. Biochem..

[91] Poona G.K., Mistry P., Raynaud F.I., Harrap K.R., Murrer B.A., Barnardb C.F.J. (1995). Determination of metabolites of a novel platinum anti-cancer drug JM216 in human plasma ultrafiltrates. J. Pharm. Biomed. Anal..

[92] Raynaud F.I., Mistry P., Donaghue A., Poon G.K., Kelland L.R., Barnard C.F.J., Murrer B.A., Harrap K.R. (1996). Biotransformation of the platinum drug JM216 following oral administration to cancer patients. Cancer Chemother. Pharmacol..

[93] Zhang J.Z., Wexselblatt E., Hambley T.W., Gibson D. (2012). Pt(iv) analogs of oxaliplatin that do not follow the expected correlation between electrochemical reduction potential and rate of reduction by ascorbate. Chem. Commun..

[94] Lasorsa A., Stuchlíková O., Brabec V., Natile G., Arnesano F. (2016). Activation of Platinum(IV) Pro-drugs by Cytochrome c and Characterization of the Protein Binding Sites. Mol. Pharm..

[95] Huang R., Zhou P.K. (2021). DNA damage repair: Historical perspectives, mechanistic pathways and clinical translation for targeted cancer therapy. Signal Transduct. Target Ther..

[96] Pace P., Mosedale G., Hodskinson M.R., Rosado I.V., Sivasubramaniam M., Patel K.J. (2010). Ku70 Corrupts DNA Repair in the Absence of the Fanconi Anemia Pathway. Science.

[97] Huang R.X., Zhou P.K. (2020). DNA damage response signaling pathways and targets for radiotherapy sensitization in cancer. Signal Transduct. Target Ther..

[98] Qin X., Fang L., Chen F., Gou S. (2017). Conjugation of platinum(IV) complexes with chlorambucil to overcome cisplatin resistance via a “joint action” mode toward DNA. Eur. J. Med. Chem..

[99] Babak M.V., Zhi Y., Czarny B., Toh T.B., Hooi L., Chow E.K.H., Ang W.H., Gibson D., Pastorin G. (2019). Dual-Targeting Dual-Action Platinum(IV) Platform for Enhanced Anti-cancer Activity and Reduced Nephrotoxicity. Angew. Chem. Int. Ed..

[100] Abdelgawwad A.M.A., Monari A., Tuñón I., Francés-Monerris A. (2022). Spatial and Temporal Resolution of the Oxygen-Independent Photoinduced DNA Interstrand Cross-Linking by a Nitroimidazole Derivative. J. Chem. Inf. Model..

[101] Kazmierczak D., Jopek K., Sterzynska K., Nowicki M., Rucinski M., Januchowski R. (2022). The Profile of MicroRNA Expression and Potential Role in the Regulation of Drug-Resistant Genes in Cisplatin- and Paclitaxel-Resistant Ovarian Cancer Cell Lines. Int. J. Mol. Sci..

[102] Krasnovskaya O.O., Akasov R.A., Spector D.V., Pavlov K.G., Bubley A.A., Kuzmin V.A., Kostyukov A.A., Khaydukov E.V., Lopatukhina E.V., Semkina A.S. (2023). Photoinduced Reduction of Novel Dual-Action Riboplatin Pt(IV) Pro-drug. ACS Appl. Mater. Interfaces.

